# Revealing and avoiding bias in semantic similarity scores for protein pairs

**DOI:** 10.1186/1471-2105-11-290

**Published:** 2010-05-28

**Authors:** Jing Wang, Xianxiao Zhou, Jing Zhu, Chenggui Zhou, Zheng Guo

**Affiliations:** 1 Bioinformatics Centre, School of Life Science and Technology, University of Electronic Science and Technology of China, Chengdu, 610054, China; 2 College of Bioinformatics Science and Technology, Harbin Medical University, Harbin 150086, China

## Abstract

**Background:**

Semantic similarity scores for protein pairs are widely applied in functional genomic researches for finding functional clusters of proteins, predicting protein functions and protein-protein interactions, and for identifying putative disease genes. However, because some proteins, such as those related to diseases, tend to be studied more intensively, annotations are likely to be biased, which may affect applications based on semantic similarity measures. Thus, it is necessary to evaluate the effects of the bias on semantic similarity scores between proteins and then find a method to avoid them.

**Results:**

First, we evaluated 14 commonly used semantic similarity scores for protein pairs and demonstrated that they significantly correlated with the numbers of annotation terms for the proteins (also known as the protein annotation length). These results suggested that current applications of the semantic similarity scores between proteins might be unreliable. Then, to reduce this annotation bias effect, we proposed normalizing the semantic similarity scores between proteins using the power transformation of the scores. We provide evidence that this improves performance in some applications.

**Conclusions:**

Current semantic similarity measures for protein pairs are highly dependent on protein annotation lengths, which are subject to biological research bias. This affects applications that are based on these semantic similarity scores, especially in clustering studies that rely on score magnitudes. The normalized scores proposed in this paper can reduce the effects of this bias to some extent.

## Background

Many scores for measuring semantic similarity (also termed functional similarity) between proteins have been proposed, based on the Gene Ontology (GO) terms [1] used to annotate the proteins. Some semantic similarity scores for a protein pair [2,3] are calculated by combining the similarity scores for the term pairs [4-7] describing the two proteins. Other scores between proteins that do not use pairwise similarity scores between terms have also been proposed [8,7-12]. Similarity scores for protein pairs have been widely applied in functional genomic research [13]. These scores are commonly used to analyze the correlation between functional similarity and similarities on other aspects, such as amino acid sequence similarity [2,8,14-16], or expression similarity [17-19]. Another type of applications is finding functional clusters of proteins [7,20-22], or functional modules in physical or genetic protein-protein interaction networks [23-28]. Similarity scores are also used to predict protein functions [29-35], protein-protein interactions [36-41] and putative disease genes [42-45].

GO protein annotations are known to be incomplete [46], and suffer from a large research bias, because certain proteins, such as those related to diseases, tend to be studied more intensively [43,47,48]. Such an annotation bias may affect protein semantic similarity scores. In this paper, we evaluated 14 common semantic similarity scores for protein pairs, and demonstrated that the scores significantly correlated with the numbers of annotation terms for the proteins (i.e., the annotation length). Thus, we proposed normalizing the scores based on their power transformation to reduce annotation bias effects, and we provide evidence that this improves performance in some applications.

## Methods

### Gene Ontology (GO)

The GO annotation data for human was downloaded from the UniProt database http://www.ebi.ac.uk/GOA/index.html, including versions in November (Nov) 2001, Nov 2002, Nov 2003, Nov 2004, Nov 2005, Nov 2006, Nov 2007 and August 2008. The GO vocabulary data were downloaded from the GO website http://www.geneontology.org in August 2008. Here, we considered only IS-A links in GO [5,6], and we mainly present the results based on the "Biological Process" (BP) sub-ontology. We also observed that all the semantic similarity scores for pairs of term groups are dependent on the annotation lengths when using "Molecular Function" and "Cellular Component" (data not shown).

### Online Mendelian Inheritance in Man (OMIM) database and disease classification

The data for 1996 genes associated with 2192 diseases were downloaded from the OMIM database [49]ftp://ftp.ncbi.nih.gov/repository/OMIM in August 2008, of which 1752 genes were annotated to GO BP terms. According to Goh et al. [50], the 2192 diseases were classified into 20 primary disorder classes based on the affected physiological systems. Diseases with multiple clinical features were assigned to the "multiple" class, and disease assigned to "Unclassified" class were not analyzed.

### Similarity scores for term pairs

Many semantic similarity scores for two proteins are based on combinations of the similarity scores for term pairs between two groups of protein annotation terms. We evaluated four semantic similarity scores for term pairs based on the information contents: Resnik score [6], Lin score [5], Relevance score (RS) [4] and Jiang score [7]. The information content of a term *c *was defined as *IC*(*c*) = -log(*p*(*c*)), where *p*(*c*) is the number of gene products annotated to the term and its descendants, divided by the number of all gene products annotated to the GO BP ontology. Let *P*(*m*, *n*) represent the set of common ancestor terms of *m *and *n*, then the four scores between terms *m *and *n *were calculated as:

### Similarity scores for protein pairs based on pairwise similarity scores between term groups

In some methods, the similarity scores for term pairs describing two proteins are combined to calculate the semantic similarity scores of the two proteins. Here, two combination methods were evaluated: the arithmetic average (AVG) of the pairwise semantic similarity scores between two groups of GO terms describing the two proteins [2] and the best-match average (BMA) approach [3].

*A_1 _*and *A_2 _*were the groups of annotation terms for proteins *P_1 _*and *P_2_*, and *#P_1 _*and *#P_2 _*were the number of terms included in *A_1 _*and *A_2_*. Then the two combined scores between the two proteins were defined as:

where , .

In total, eight semantic similarity measures for protein pairs were evaluated, using the four semantic similarity scores for term pairs (Resnik, Lin, RS and Jiang) combined with the AVG and BMA methods (see Table 1).

**Table 1:** Summary of 14 semantic similarity scores for protein pairs.

Measure	Description	Range
Similarity scores for term pairs		

Resnik [6]	Information content of the most informative common ancestor of two terms	≥ 0
Lin [5]	Normalized Resnik similarity score by assessing how close two terms are to their most informative common ancestor	[0, 1)
RS [4]	Weighted Lin similarity score by using the probability of annotations of the most informative common ancestor	[0,1)
Jiang [7]	Based on the difference between two terms and their most informative common ancestor in information content	(0,1]

Similarity scores for protein pairs based on pairwise similarity scores between term groups		

AVG [2]	The average of the similarity scores for all pairs of terms between two groups of protein annotations	Same with those for the corresponding similarity scores for term pairs
BMA [3]	The score of the best-matching pairs between two groups of protein annotations	

Similarity scores for protein pairs based on groupwise similarity scores between term groups		

TO [9]	The number of terms shared by the annotations for two proteins	≥ 1
NTO [9]	Dividing TO by the minimum of the annotation lengths of two proteins	(0,1]
Dice [12]	Dividing TO by the average of annotation lengths of two proteins	(0,1]
Kappa [11]	A chance-corrected measure of co-occurrence between two groups of protein annotations	[0, 1]
GIC [8]	Jaccard index weighted by the information content of each GO term	[0, 1]
VSM [10]	Cosine similarity weighted by the information content of each GO term	[0, 1]

### Similarity scores for protein pairs based on groupwise similarity scores between term groups

We also evaluated six protein semantic similarity scores that do not use pairwise similarity scores between two term groups. These similarity scores are briefly described as below (please see details in the original papers).

(1) The TO (Term Overlap) score [9] simply counts the number of overlapped terms for two proteins *P_1 _*and *P_2 _*as follows:

where *GA*_1 _and *GA*_2 _include the terms directly annotated with proteins *P_1 _*and *P_2 _*and all their ancestral terms, respectively. #(*GA_1 _*∩ *GA_2_*) is the number of the overlapped terms between *GA_1 _*and *GA_2_*.

(2) The NTO (Normalized Term Overlap) score [9] is defined as:

(3) The Dice score [12] is defined as:

(4) The Kappa score [11] is defined as:

where  and  represent the observed and random co-occurrence of GO annotation terms for the two proteins, respectively.

(5) The Graph Information Content (GIC) score [8] is defined as:

(6) The Vector Space Model (VSM) score [10] is defined as follow:

where *n *is the number of all the GO BP terms and  is the information content of term *k *if it is annotated with protein *P_1 _*(*P_2_*) while  is 0 if the term *k *is not an annotation of the protein *P_1 _*(*P_2_*).

In total, we evaluated 14 semantic similarity scores for protein pairs (see Table 1). We note that some other semantic similarity scores for protein pairs [13,51] were not evaluated in this paper. For example, the score proposed by Wang et al. [22], which weights the IS-A and PART-OF links of GO, was not analyzed, because we considered only IS-A links in this study.

### Random experiments

Using randomly selected pairs of term groups, we evaluated the increase in protein semantic similarity score that resulted from only the increased annotation length, regardless of other biological factors. First, we randomly selected 10,000 pairs of term groups with the same sizes (corresponding to the annotation lengths of proteins) ranging from 1 to 10, since only 1.5% of proteins had more than 10 annotations in GO BP ontology. Then, using each of the 14 semantic similarity scores described above, we calculated the semantic similarity scores for random term group pairs, and analyzed whether these scores increased as the group size increased using the Spearman rank correlation coefficient [52].

### Normalization based on power transformation

As demonstrated in the *Results *section, a similarity score for two groups of terms is dependent on the lengths of the term groups. To reduce the effect of the lengths on the scores, we took two steps to make the scores for pairs of term groups with given length combinations follow the standard normal distribution.

Firstly, we applied the commonly used power transformation approach to transform the scores to achieve normality [53,54]. Suppose *SS*(*TG*_1_, *TG*_2_) is the score for term groups *TG_1 _*with length *L_1 _*and *TG_2 _*with length *L_2_*, we power-transformed it to *TSS*(*TG*_1_, *TG*_2_) = . Here, the power  was estimated as follow [53,54]:

where *M*_*SS *_is the median of the random *SS*(*TG*_1_, *TG*_2_) distribution which was estimated by the similarity scores for 10,000 pairs of random term groups (with lengths *L_1 _*and *L_2_*). *T*_*q *_and *T*_1-*q *_are the lower and upper *q*th quantiles of this distribution (). By the one-sample Kolmogorov-Smirnov test for distribution goodness-of-fit [55], at the significance level of 0.1, we tested whether the power-transformed scores for pairs of term groups with given length combinations fit normal distributions.

Secondly, we normalized the above power-transformed scores to make them fit the standard normal distribution as follow:

In the above normalization formula, *M*_*TSS *_and *STD*_*TSS *_are the median and standard deviation of the power-transformed scores respectively. Here, we used the median rather than the mean in the normalization formula because it might be more appropriate for measuring the location parameter of a distribution when the distribution might be skewed [56-58]. As shown in the *Results *section, most of the normalized scores for pairs of term groups with given length combinations follow normal distributions. In this situation, the means and the medians are equal.

### Sequence similarity scores for protein pairs

Amino acid sequence data for human proteins was downloaded from UniProt ftp://ftp.uniprot.org in August 2008. The sequence similarity between two proteins was measured by the ln(bit score), and calculated by the NCBI "blastall" program [2]. Sequence similarity scores were obtained for a total of 499,878 protein pairs with GO BP annotations.

### Clustering algorithm and enrichment analysis

Suppose the original and normalized similarity scores for two proteins (*P_1 _*and *P_2 _*) annotated with two groups of terms are *SS*(*P*_*1*_, *P*_*2*_) and *NSS*(*P_1_, P_2_*) respectively, we firstly transformed both *SS*(*P_1_, P_2_*) and *NSS*(*P_1_, P_2_*) to the range [0, 1] by the Min-Max normalization method [59,60] as follows

where *Max_SS _*and *Min_SS _*are the maximum and minimum values of the original similarity scores for all protein pairs from a protein set (e.g., a set of proteins encoded by a set of disease genes). *Max_NSS _*and *Min_NSS _*are the maximum and minimum values of the normalized similarity scores for all these protein pairs.

Then, we calculated the distance between two proteins as *D*(*P_1_*, *P_2_*) = 1-*MM*(*P_1_*, *P_2_*) based on the original score. Similarly, based on the normalized score, the distance was calculated as *ND*(*P_1_*, *P_2_*) = 1-*NM*(*P_1_*, *P_2_*). Both *D*(*P_1_*, *P_2_*) and *ND*(*P_1_*, *P_2_*) take values ranging from 0 to 1 and satisfy three main properties of distance metrics [61]: (i) symmetry, *D*(*P_1_*, *P_2_*) = *D*(*P_2_*, *P_1_*) (*ND*(*P_1_*, *P_2_*) = *ND*(*P_2_*, *P_1_*)); (ii) non-negative, *D*(*P_1_*, *P_2_*) ≥ 0 (*ND*(*P_1_*, *P_2_*) ≥ 0); (iii) triangle inequality, *D*(*P_1_*, *P_3_*) ≤ *D*(*P_1_*, *P_2_*)+*D*(*P_2_*, *P_3_*) (*ND*(*P_1_*, *P_3_*) ≤ *ND*(*P_1_*, *P_2_*)+*ND*(*P_2_*, *P_3_*)). Using *D*(*P_1_*, *P_2_*) and *ND*(*P_1_*, *P_2_*) respectively, we clustered disease genes by the complete linkage clustering algorithm [62].

To evaluate the clustering results, using the hypergeometric distribution model [63,64], we calculated the probability *p *of detecting at least the observed number of genes related to a disease category proposed by Goh et al. [50] in a cluster of disease genes by random chance. The *p *values were corrected by the false discovery rate (FDR) by the Benjamini-Hochberg (BH) procedure [65]. With FDR of 1%, we found the disease categories enriched in a cluster of disease genes found by the clustering algorithm.

## Results

### The dependence of the semantic similarity scores on annotation lengths

**Figure 1:**
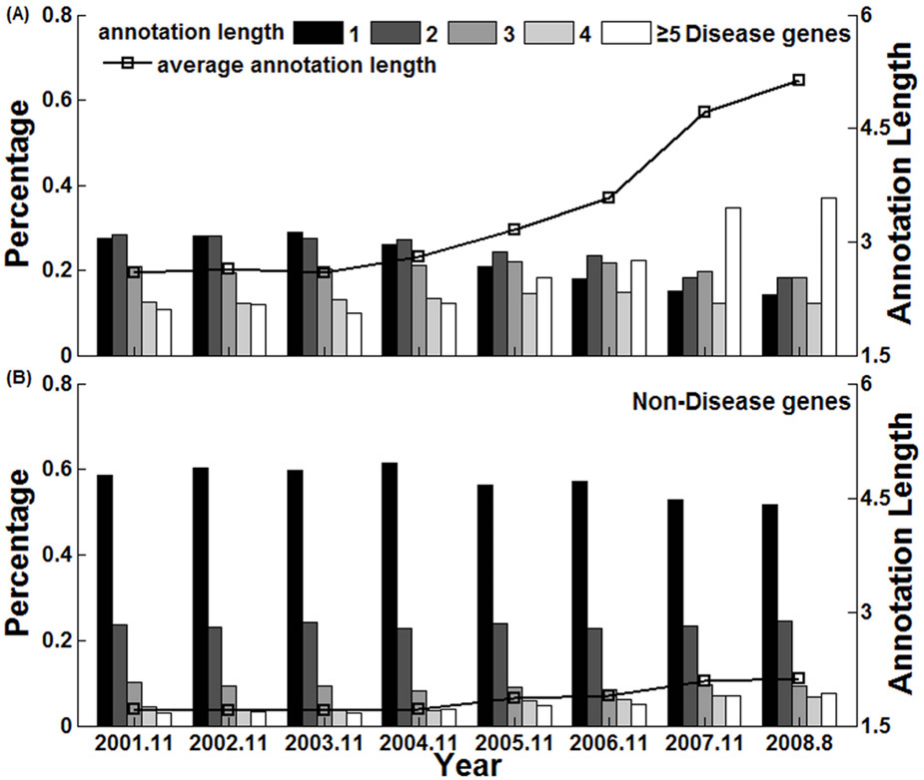
**Changes of the gene annotation lengths in GO BP ontology**. The bar plots represent the percentages of disease and non-disease genes with different annotation lengths, while the solid-square lines represent the average annotation lengths in various versions of GO.

From 2001 to 2008, the average number of GO BP terms annotated with disease genes increased from 2.6 to 5.1, as shown in Figure 1(A). In contrast, the average annotation length of "non-disease" genes increased slightly from 1.7 to 2.1 (Figure 1(B)). These results indicated that disease genes tend to be studied more extensively, and are biased to have more annotations.

**Figure 2:**
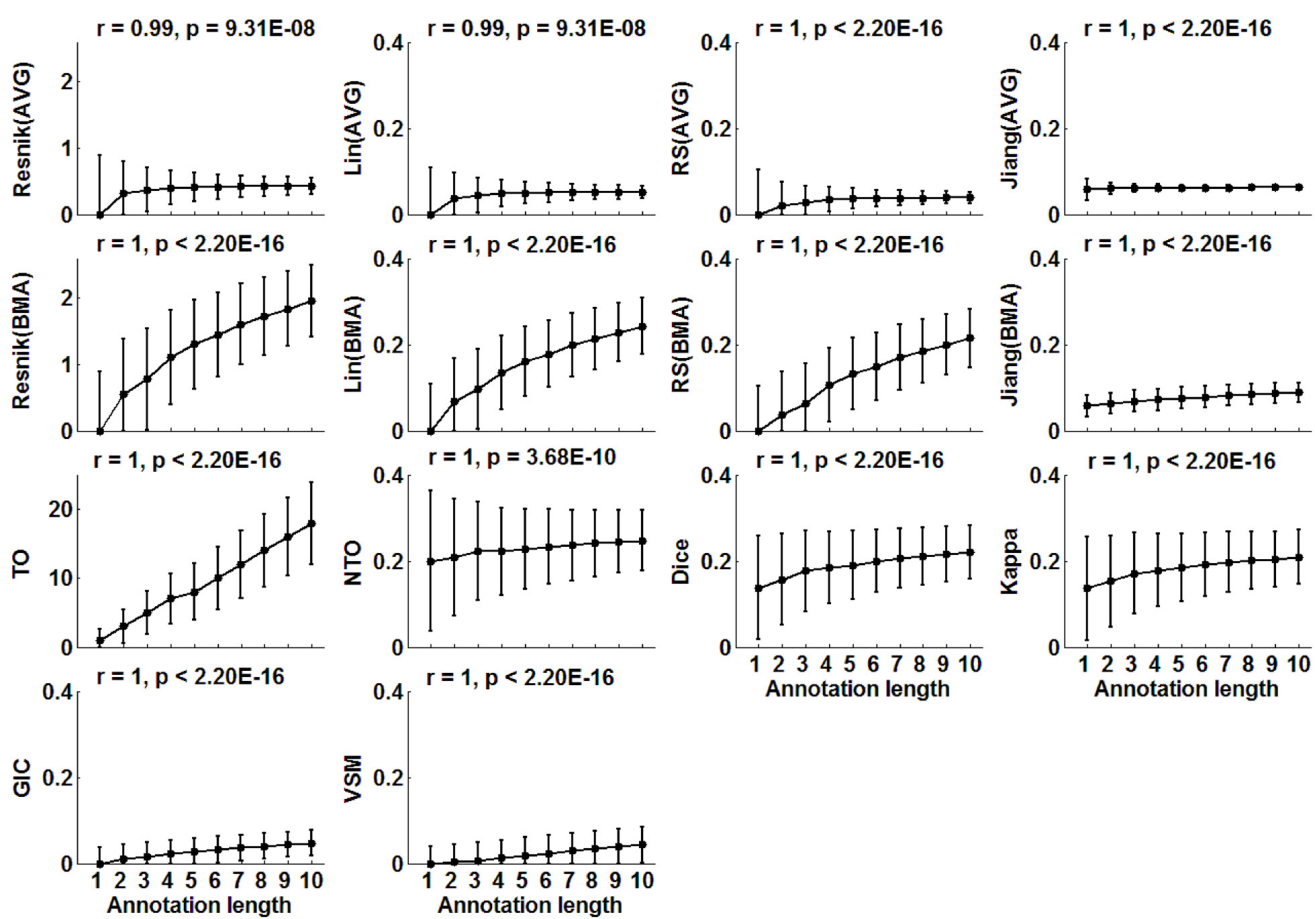
**Semantic similarity scores for pairs of term groups increase as the annotation lengths increase**. For each of the 14 semantic similarity measures, the solid circles represent the median score of 10,000 random pairs of term groups annotated with the same number of GO BP terms. The error bars represent the standard variation of the scores (because the similarity scores were positive, we reduced the negative part of error bars). *r *and *p *are the Spearman correlation coefficient and the corresponding significance between the median of the scores and the annotation lengths. To make the 14 plots more informative, according to the ranges of the scores (see Table 1), we classify the plots into three groups, in each of which the plots have the same y-axis scale.

As shown in Figure 2, for each of the 14 protein semantic similarity scores analyzed, the median score for 10,000 random pairs of term groups increased significantly as the annotation lengths (the group sizes) increased (Spearman *r *≥ 0.99, *p *< 1E-07). Based on the Resnik [6], Lin [5], RS [4] and Jiang [7] semantic similarity scores for term pairs, all four AVG combined scores for protein pairs were relatively stable when the annotation length was greater than three, especially for the Jiang(AVG). In contrast, the combined scores based on Resnik(BMA), Lin(BMA) and RS(BMA) increased rapidly with the annotation length. We evaluated six other semantic similarity scores for protein pairs, which do not use pairwise similarity scores between terms. As shown in Figure 2, the TO score was linearly dependent on the annotation length, while other scores increased slightly but significantly as the annotation length increased (Spearman *r *= 1, *p *< 1E-09). Notably, as shown in Figure 2, as the annotation lengths increased, the standard deviation of TO scores increased but it decreased for other similarity scores, which could be explained statistically. For example, because TO scores follow the hypergeometric probability model [63,64], we can derive that its standard deviation increases with the annotation lengths.

### Applications of the normalized scores

As shown in Table 2, based on each of the 14 similarity measures for term groups, most of the original scores (*SS*(*TG*_1_, *TG*_2_)) for pairs of term groups with given length combinations did not fit normal distributions (*p *≥ 0.1, one-sample Kolmogorov-Smirnov test). For nine similarity measures, namely the Resnik(AVG), Resnik(BMA), Lin(AVG), Lin(BMA), RS(AVG), RS(BMA), Jiang(AVG), Dice and Kappa scores, over 80% of the power-transformed scores for pairs of term groups with given length combinations followed normal distributions. Then, these power-transformed scores were normalized to the standard normal distribution. Thus, for these nine similarity measures, the normalization method based on the power transformation is largely suitable for comparing scores for pairs of term groups with different length combinations. However, based on each of the other five similarity measures, less than 60% of the power-transformed scores fitted normal distributions. We also analyzed another five simple transformation methods and the results (see Table 2) showed that all these simple transformation methods performed worse than the power-transformation method using the estimated .

**Table 2:** The performance of different data transformation methods*.

Measure	Estimated λ**	λ = 1	Inverse (λ = -1)	Cube-root (λ = 1/3)	Square-root (λ = 1/2)	Square (λ = 2)	Log
Resnik(AVG)	0.878	0	-***	0.645	0.370	0	-
Lin(AVG)	0.890	0	-	0.659	0.474	0	-
RS(AVG)	0.925	0	-	0.632	0.355	0	-
Jiang(AVG)	0.812	0	0.081	0	0	0	0.002
Resnik(BMA)	0.938	0.661	-	0.025	0.248	0	-
Lin(BMA)	0.940	0.706	-	0.012	0.156	0.002	-
RS(BMA)	0.927	0.650	-	0.004	0.042	0.001	-
Jiang(BMA)	0.010	0	0	0	0	0	0
TO	0	0	0	0	0	0	0
NTO	0.555	0.001	0	0.366	0.478	0	0.009
Dice	0.926	0.014	0	0.384	0.890	0	0.001
Kappa	0.896	0.010	-	0.518	0.866	0	-
GIC	0.552	0	-	0.096	0	0	-
VSM	0.291	0	-	0.006	0	0	-

* The numbers in the table represent the percentages of the scores that fitted normal distributions after data transformation, among all group pairs with different length combinations.

** λ was estimated by the method described in the *Methods *section.

*** "-" indicates the transformation method was not suitable for the similarity measure.

**Figure 3:**
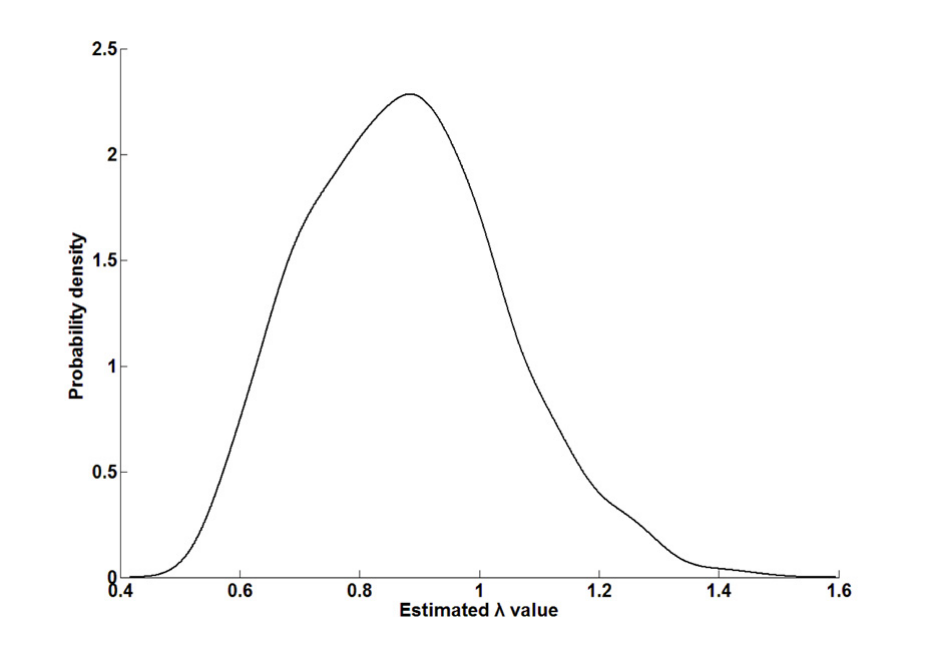
**Probability density of  estimated for pairs of term groups with different annotation length combinations based on the RS(BMA) measure**.

**Figure 4:**
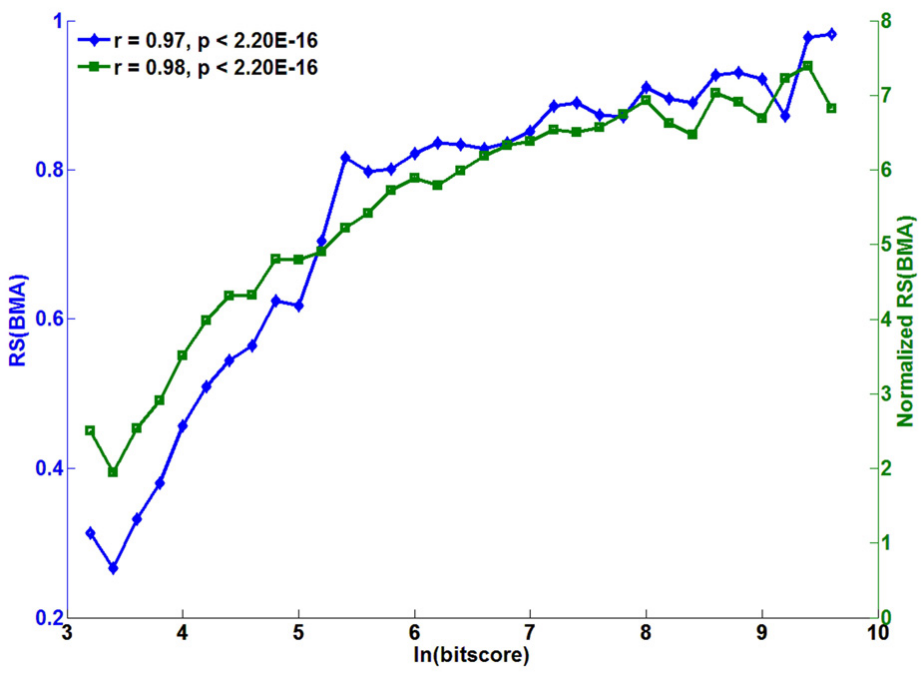
**Relationship between sequence similarity and semantic similarity of protein pairs**. The semantic similarity scores of protein pairs were calculated by RS(BMA) (blue) and normalized RS(BMA) (green) measure. The results showed that the original and normalized RS(BMA) scores had similar correlation with sequence similarity scores, and confirmed that proteins with similar sequences tend to have higher "functional similarity" [2,14].

Then, for two types of applications, by comparing the results based on the original scores and their corresponding normalized scores, we showed that the bias affects certain analysis more than others. One type of applications based on semantic similarity scores for protein pairs study the correlation between functional similarity and similarities on other aspects [2,8,14-19]. Based on the normalized RS(BMA) score (the corresponding  distribution for this measure was shown in Figure 3), we analyzed the correlation between protein semantic similarity scores and their amino acid sequence similarity scores (ln(bit score)). As shown in Figure 4, the correlation was significant (*p *< 2.20E-16), supporting the model that proteins with similar sequences tend to be functionally similar [2,14]. Based on the RS(BMA) scores, similar results were observed, because of significant correlation between the ranks of the RS(BMA) scores and the normalized RS(BMA) scores for protein pairs (Spearman *r *= 0.88, *p *< 2.20E-16).

**Figure 5:**
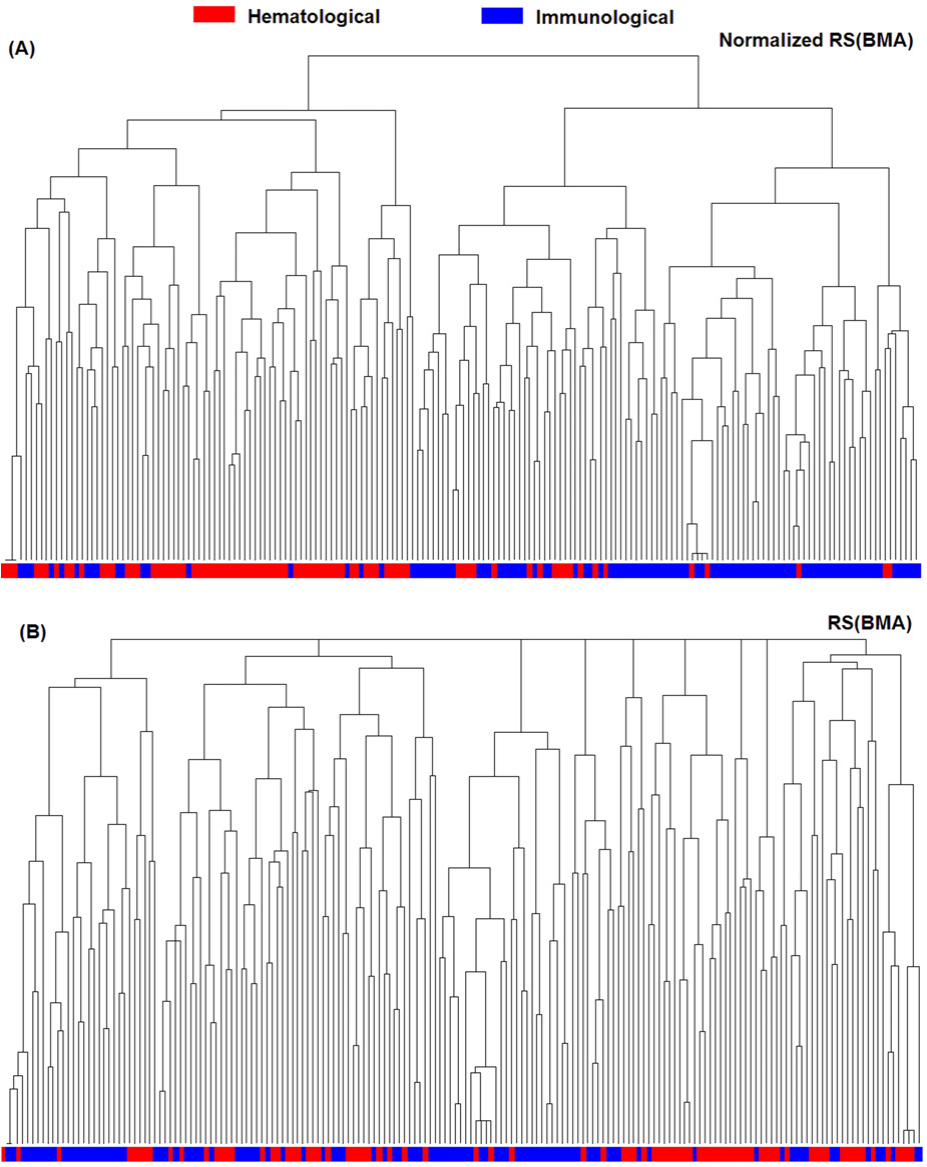
**Cluster analysis of genes in "Hematological" and "Immunological" disease categories**. Two types of disease genes were clustered by the normalized (A) and original (B) RS(BMA) methods. Horizontal axis represents all disease genes of "Hematological" (red) and "Immunological" (blue) categories.

**Figure 6:**
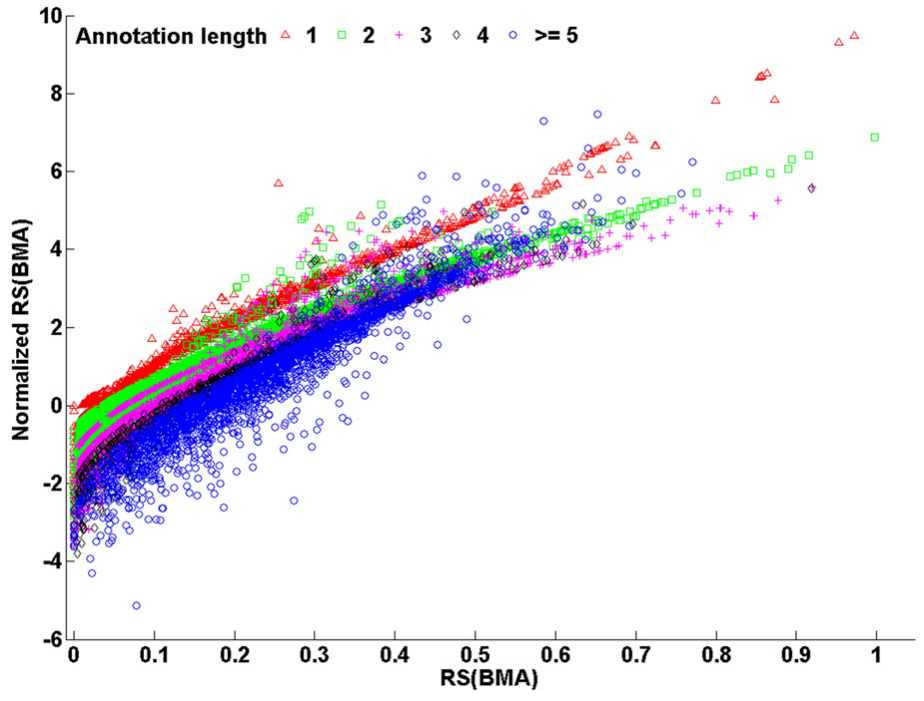
**Scatter plot of RS(BMA) vs. normalized RS(BMA) scores for pairs of genes in "Hematological" and "Immunological" disease categories**. Disease gene pairs were classified into five groups, according to the minimum of annotation lengths of the two genes: one (red triangles), two (green squares), three (magenta plus signs), four (black diamonds) and more than four (blue circles).

Another type of applications is clustering of functionally similar proteins [7,20-22] or finding functional modules in physical or genetic protein-protein interaction networks [23-28]. Using the RS(BMA) and the normalized RS(BMA) distance, we applied a hierarchy clustering algorithm to cluster the disease genes into 21 categories, and compared the results with the categories determined by Goh et al. [50]. As evaluated by the hypergenomic distribution test, using FDR of 1%, 16 clusters based on the normalized distance were enriched with disease genes with the same or similar phenotypes while only 6 clusters were enriched based on the original distance. To analyze more clearly the effect of annotation length on the cluster results, we clustered only the genes determined to the "Hematological" and "Immunological" categories. As shown in Figure 5(A), based on the normalized RS(BMA) distance, 73.5% of "Hematological" genes (red) were clustered into one group (*p *= 7.3E-13), while 78.4% of "Immunological" genes (blue) were in another (*p *= 3.96E-13). In contrast, as shown in Figure 5(B), when clustering these two classes of disease genes into two groups based on the RS(BMA) distance, no group was significantly enriched with a class of disease genes (*p *> 0.10). As shown in Figure 6, after normalization, the ranks of some disease gene pairs with different annotation lengths changed, improving the clustering results based on the normalized RS(BMA) distance. In general, based on the normalized RS(BMA) scores, our results suggested that disease genes related to the same or similar diseases tend to work together in the same disease-related functional gene modules [50,66-78].

## Discussion

In this paper, we found that most semantic similarity scores for protein pairs increased as protein annotation lengths increased. Because protein annotations are likely to be subject to biological research bias, most applications based on current semantic similarity scores for protein pairs will be biased. Without the annotation bias, one could argue that over-annotated proteins might be more likely to be similar than under-annotated proteins, when considering only shared functions, and disregarding differences. However, currently, most semantic similarity scores for protein pairs evaluate the overall functional similarity between proteins. Depending on the available knowledge about domains, and the final aim of the application, different criteria could be used to define similarity between proteins. We note that protein annotations in GO for most model organisms (e.g., *Saccharomyces cerevisiae*) are also incomplete and suffer from the research bias because important genes such as the homologues of human disease genes tend to be studied more intensively [79,80]. By analyzing the *Saccharomyces cerevisiae *data, we also found that the similarity scores between two groups of terms increased significantly with the annotation lengths (data not shown). Thus, our conclusion on the bias of semantic similarity scores for proteins would be applicable to other organisms.

A protein is usually annotated to a group of GO terms. Often, the semantic similarity scores between two proteins are calculated using some combination methods [2,3] based on the semantic similarity scores for pairs of terms annotated with the two proteins. Many semantic similarity scores for term pairs such as the Resnik [6], Lin [5], Relevance (RS) [4] and Jiang [7] are based on the information content (related to the annotation specificity) of the terms. Based on these similarity scores for term pairs, the similarity scores for two proteins might not always increase, if the proteins have many annotations with low-specificity. However, as shown here, all the AVG and BMA scores for protein pairs based on the Resnik, Lin, RS and Jiang scores for term pairs still significantly correlated with the protein annotation lengths.

To reduce the effects of protein annotation bias, we normalized the scores based on the power transformation by estimating power . The normalization method based on the power transformation can transform most scores based on nine of the similarity measures to fit normal distributions but it performs poorly for the other five similarity measures. Thus, future works are needed to further improve the data transformation and normalization method.

The feasibility of the normalized scores was analyzed for two types of applications and the results showed that the normalized scores were useful in these applications. Analysis of the correlation between functional similarities and similarities on other aspects [2,8,14-19] might be less affected by the annotation bias, because the ranks of semantic similarity scores for protein pairs and their corresponding normalized scores were highly correlated. Our results also showed that clustering analysis [7,20-22] using the magnitude of the semantic similarity scores might be more seriously affected by biased protein annotations, and the results could be improved by using the normalized scores.

A third type of applications that uses protein semantic similarity scores is predicting protein functions [29-35], protein-protein interactions [36-41] and disease genes [42-45]. However, because many other factors, such as the selection of algorithms and the definition of positive and negative sets [81] can affect the prediction results, we did not evaluate the effect of the annotation bias on these uses. Nevertheless, because this type of applications also uses the similarity score magnitudes, we recommend also using normalized scores in prediction studies, to reduce the effects of the annotation bias.

To avoid the influence of annotation bias, other approaches may be attempted. For example, the statistical *p*-value of a semantic similarity score for a protein pair could be evaluated by comparing this score with the scores of random protein pairs with the same annotation lengths. If the semantic similarity score of the two proteins was significantly larger than the score expected by random chance, at a given significance level (*p*-value), we could determine that the two proteins are functionally similar [82]. Functional modules could be found by linking functionally related proteins. To analyze the functional relationships of proteins more comprehensively, the semantic similarity scores should be combined with other functional data, such as protein-protein interaction, co-expression and co-conservation of proteins [83-85].

## Conclusions

Current protein semantic similarity scores are highly dependent on protein annotation lengths, which are subject to biological research bias. This bias may affect many current applications based on these scores. The proposed normalization method might solve this problem to some extend.

## Authors' contributions

Conceived and designed the experiments: ZG, JW. Performed the experiments: JW, XZ and CZ. Analyzed the data: ZG, JW and JZ. Wrote the paper: JW, JZ and ZG. All authors read and approved the final manuscript.
